# A Novel Method to Measure the Static Coefficient of Friction for Socks

**DOI:** 10.3390/s22155525

**Published:** 2022-07-25

**Authors:** Jinsu Eun, Jaejin Ryue, Sangsoo Park, Kikwang Lee

**Affiliations:** 1 College of Physical Education, Kookmin University, Seoul 02707, Korea; eunjinsu123@kookmin.ac.kr (J.E.); jjryue@k2korea.co.kr (J.R.); 2 Dooree System Technology Co., Ltd., Seoul 13219, Korea; 3 Footwear R&D team, K2 Korea Co., Ltd., Seoul 06373, Korea; 4 College of Medicine, Korea University, Seoul 02841, Korea

**Keywords:** ramp test, last, non-slip socks

## Abstract

Mechanical testers have commonly been used to measure the frictional properties of socks. However, the friction values may be susceptible to the level of stretchiness of tested fabrics or human variability. Thus, the aim of this study was to propose a novel method that enables friction measurement of socks in a sock-wearing condition with less human variability effects. Five socks with different frictional properties were chosen. Three experimental ramp tests were performed with an artificial structure shaped like the foot-ankle complex (last) and a ramp tester to quantify the static coefficient of friction (COF) at the foot against sock, at the sock against an insole, and the foot wearing socks against the insole, respectively. The angle where the last slipped while the ramp surface was gradually inclined was used to compute the static COF values for each sock. The reliability was 0.99, and COF values ranged from 0.271 to 0.861 at the foot-sock interface, 0.342 to 0.639 at the sock-insole interface, and 0.310 to 0.614 in the third test. Socks with different frictional properties were successfully distinguished each other. Thus, the suggested protocol could be a reliable option for measuring the static COF values in the tension similar with it found in a sock-waring condition with reduced effects of human variability.

## 1. Introduction

Socks can alter frictional properties at the foot-insole interface. Wearing socks help maintain body temperature and manage the hydration level of foot skin. Besides the physiological body protection functions, socks may reduce the risk of blisters by lowering the friction between the feet and insole [1,2,3]. However, too low friction at the interface can cause slip on the insole [1,4], suggesting that proper frictional properties are critical for preventing falling in daily life [5] and preventing a decline in performance in sports due to pain triggered by blisters [6]. Socks can provide friction at the foot skin against the inner and outer parts of socks against the shoe insole, but their friction measurements have been challenging since the interfaces are invisible. The outer part of the shoe surrounding the foot prevents observing the foot motion on the shoe insole. One indirect method of estimating the frictional properties was to use mechanical testers involving a specific size of sample fabric and human participants [7,8,9,10,11,12,13,14]. However, the fabric tension on the testers may not represent the sock tension originating from the stretchiness in an actual sock-wearing condition. Furthermore, the method often requires recruiting human participants and providing them with a practice session for mastering proper force production by their foot on the surface of sample fabrics, which may cause significant efforts for performing the testing and the results to be susceptible to human variability. Therefore, there has been a need to develop a time-efficient method that enables the measurement of frictional properties in a sock-wearing situation without the involvement of human participants. 

Sock stretchiness and human variability could be important factors that should be considered when the frictional properties of socks are measured. When we wear socks, socks are stretched and make full contact along the foot shape. To imitate the tension originating from the stretchiness, a certain size of fabric was stretched and then hung in mechanical testers, but it remained unclear how close the pre-tension levels were to the actual tension occurring in a sock-wearing condition. Previous studies have shown that the pre-tension magnitudes could cause changes in COF values at contact interfaces [15,16]. COF values were increased as pre-tension magnitudes of testing fabrics were increased [16], suggesting that more real-world friction measurements should include the stretchiness occurring in the sock-wearing condition. Human participants have often been recruited for measuring frictional properties at the skin-fabric interface. The friction at the skin-fabric interface has been measured by the ratio of tangential to normal forces produced on sock samples by either human participants [11,13,17] or a probe moved on the skin [11]. However, variability of forces produced by human participants [18] may interrupt more precise measurement of friction at the contact interface. Using a dummy shaped like the foot-ankle complex (last) would be able to better replicate the tension [19], but it has still required force production by humans. 

A ramp tester with the last may be an alternative option to measure the friction of socks without human participants. The ramp test protocol has been known as a reliable option to measure the friction at the outsole and flooring [20,21,22]. The ramp test is initiated by setting a flooring condition (e.g., water) on the ramp surface and requesting a human participant to stand up on that surface while wearing a testing shoe, followed by a gradual increase in the inclination angle of the surface [20]. Then, the angle when participants lost their balance was used to measure the coefficient of friction at the outsole-flooring interface. Although the ramp test protocol has been reliable and outperformed other methods using mechanical devices for predicting fall risk [21], the protocol still requires human participants, which could result in similar issues observed in the previous studies using mechanical testers [7,8,11]. This disadvantage may be overcome by using a dummy [19,23] that can replace human participants. However, there have not been such studies that have examined whether the ramp protocol with the last can be used to measure frictional properties at the contact interfaces of the foot, sock, and insole.

Thus, this study aimed to develop a novel method to measure static COF values at both the skin-sock and sock-insole interfaces in a wearing condition with minimal effects of human variability. An artificial structure (last) shaped like the human ankle-foot complex was developed and was worn socks to imitate sock-wearing conditions observed in daily life. The last was placed on the surface of a ramp tester [20], and the surface was gradually inclined. The slip angle when the last started to move was used to compute the static COF for socks in this study. Five socks, including stockings, were chosen to evaluate the proposed method. We hypothesized that the suggested method successfully identifies the differences in frictional properties of socks. 

## 2. Materials and Methods

### 2.1. Types of Socks

**Figure 1:**
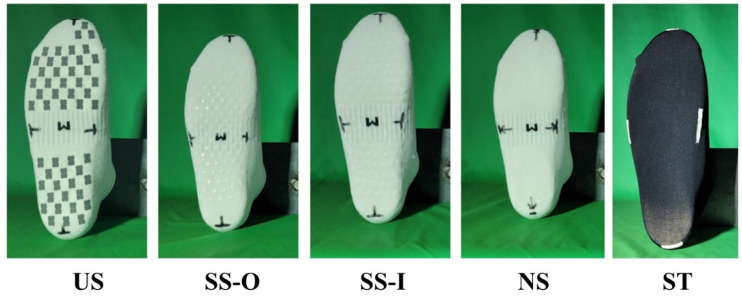
Five socks tested in this study (US, Socks with polyurethane non-slip pads; SS-O, socks with silicone protrusions attached to the outer surface; SS-I, socks with silicone protrusions attached to the inner surface; NS, regular socks without any non-slip materials attached; ST, stockings).

Five types of socks were selected to evaluate their non-slip function. There were socks with polyurethane non-slip pads attached on both inner and outer parts of the socks (US), socks with silicone protrusions attached to the outer part (a part contacts with the insole; SS-O), socks with silicone protrusions attached to the inner part (a part contacts with foot skin; SS-I), regular socks without any non-slip materials attached (NS), and stockings (ST) (Figure 1). Except for ST, the composition materials, ratios, and sock shape were the same for all socks (cotton, polyester, and polyurethane; WE FOOT TECHNOLOGY, Seoul, Korea). ST was composed of nylon and polyurethane.

### 2.2. The Mechanical Ramp Tester and Last

**Figure 2:**
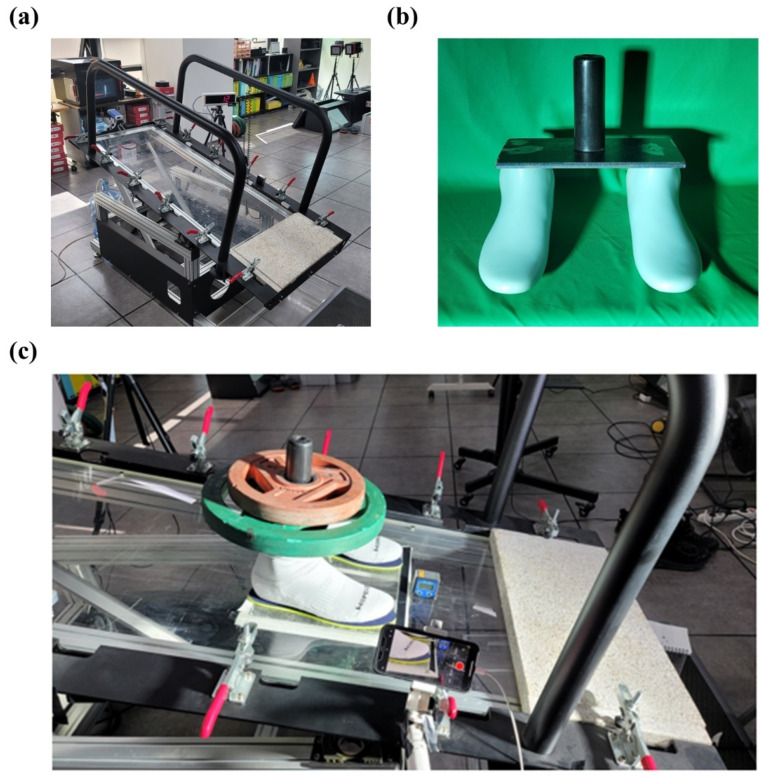
The experimental setup for the ramp protocol. (**a**) A foot-shaped structure (last, 5 kg) was developed to mimic the actual sock-wearing condition in the friction tests; (**b**) we added two round-shaped disks (15 kg) on the metal plate of the last in the experiment; (**c**) slip angle was defined as the angle when both feet of the last crossed over the black-colored tape (width: 2 cm). The slip angle was measured by the inclinometer.

We developed a mechanical ramp tester of which the surface inclination angle can be controlled (Figure 2a). A potentiometer (COSMOS RV24YN 20S B101, Tokyo Cosmos Electric Co., Sobubai, Zama, Kanagawa, Japan) was installed at the rotational axle of the ramp surface to measure and control its inclination angle (range = 0° to 60°, resolution = 1°). The surface was made of acrylic so that diverse test materials can be attached. A dummy foot-ankle complex (last) was utilized in the study (Figure 2b). The structure last consisted of a pair of 260 mm foot models interspersed with 20 cm welded and bonded to a metal plate (width: 12 cm, length: 27 cm, height: 1 cm). A metal cylinder (height: 14 cm, diameter: 4.5 cm) was rigidly attached to the middle of the medal plate. The combined mass of the plate and the cylinder was 5 kg. Two round-shaped weights (10 kg and 5 kg for each) were placed on the last (20 kg; Figure 2c). An inclinometer (range = 0° to 360°, resolution = 0.1°; AG-0200BB, Bevel Box, Gain Express Holdings Ltd., To Kwa Wan, Hong Kong) was attached on the ramp surface to measure the angle of the surface inclination. A smartphone (iPhone 12, Apple, Los Altos, CA, USA) was attached to the edge of the surface to record both last movement at the sagittal plane and an angle value shown from the inclinometer (30 Hz; Figure 2c). 

### 2.3. A Ramp Protocol to Measure the Static Coefficient of Friction

**Figure 3:**
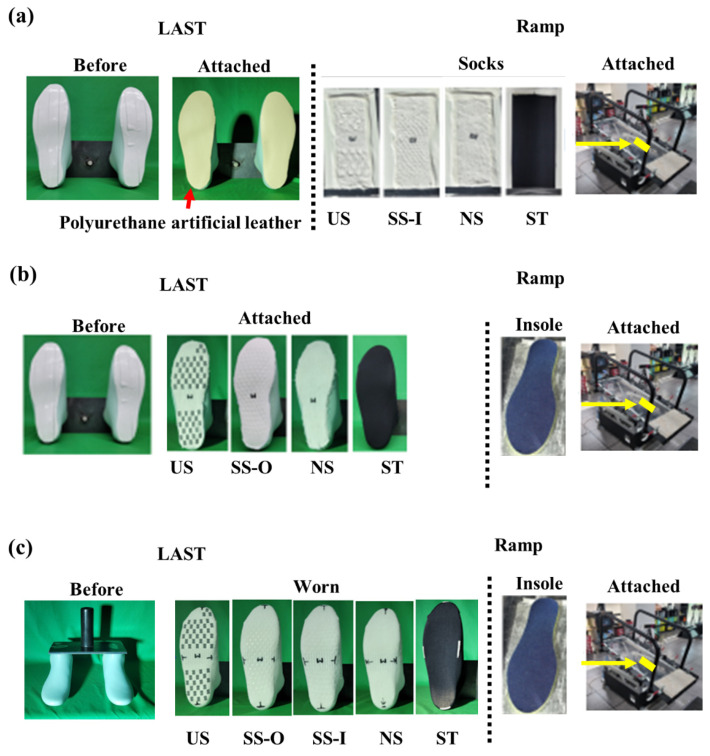
Three ramp conditions. (**a**) a set-up to measure the static COF at the skin-sock interface; (**b**) a set up to measure the static COF at the sock-insole interface; (**c**) a setup to measure the static COF at the sock-insole interface in an actual wearing scenario.

The static coefficient of friction (COF) for each sock was measured by a ramp test protocol that has been used to determine friction at the foot-floor surface [5,10,20,24]. We gradually increased the inclination angle of the ramp by a speed of around 0.6°/s and the angle where both feet of the last crossed over the attached tape (width: 2 cm) was measured by reading the angle value on the inclinometer in the recorded videos. Three experimental ramp tests were performed to quantify the static coefficient of friction at the foot against the sock, the sock against an insole, and the foot wearing socks against the insole (Figure 3). The first ramp condition was performed to measure the coefficient of friction (COF) between the inner part of the sock and the foot skin (Figure 3a). Human foot skin was represented by artificial polyurethane leather that has shown to be suitable for imitating the frictional characteristics of human skin due to the similarity of roughness and surface [25]. The artificial leather was cut into a shape of the bottom of the last and attached at its bottom while a sample of the inner part of each sock was attached to the surface of the mechanical ramp. Four socks excluding SS-O were used in the first ramp condition since the inner part of SS-O was the same as one of NS. The static COF values at the outer part of the sock against the shoe insole were measured for each sock type in the second ramp condition (Figure 3b). To imitate the stretchiness in sock-wearing situations, socks were cut into the last foot shape while the last was worn the socks. A shoe insole from commercial shoes (280 mm; Rote Rivre FL5, ASICS Korea, Seoul, Korea) was attached to the surface. SS-I was excluded as it had the same outer part as did NS. In the third ramp condition, the last was worn each sock and was placed on the same insole attached to the surface (Figure 3c). The last experiment measured the static COF at the sock-insole interface that imitated the resultant interfaces (foot-sock-insole) in the real-wearing condition. In each experimental ramp condition, seven trials were performed for each sock. The ramp surface was cleaned before each trial, and the attached sock or insole status was checked immediately before and after each trial for consistency. Each sock was tested in a relatively small number of measurements for a relatively short duration so that the effects of changes in its frictional properties by the stretchiness on the friction measurements may be minimal.

After excluding the maximum and minimum angle values [20], the remaining five slip angle measurements were used to compute the coefficient of friction using

(1) $$\mu =\frac{\sin\theta }{\cos\theta }=\tan\theta$$

where μ is the coefficient of friction and θ is the slip angle. 

### 2.4. Statistical Analyses

Inter-trial reliability between five COF values measured by the ramp test was quantified using the intraclass correlation coefficient (ICC). Reliability was classified as slight (0.00–0.20), fair (0.21–0.40), moderate (0.41–0.60), substantial (0.61–0.80), and almost perfect (>0.80) [26]. For each the ramp conditions, a one-way ANOVA was performed for each ramp condition to compare the static COF values. The Greenhouse-Geisser *p*-value adjustment was performed when the sphericity assumption was not satisfied. Following pairwise comparisons with Bonferroni adjustment were performed when the main effect was significant. All the statistical analyses were performed in SPSS statistical software (V26, IBM, Armonk, NY, USA), and the level of significance was set to *p* < 0.05. 

## 3. Results

The intraclass correlation coefficient was computed to evaluate the inter-trial reliability of static COF measurements (Supplemental Table S1). The COF measurement reliability was 0.998 (95% CI [0.995–0.999]). 

**Figure 4:**
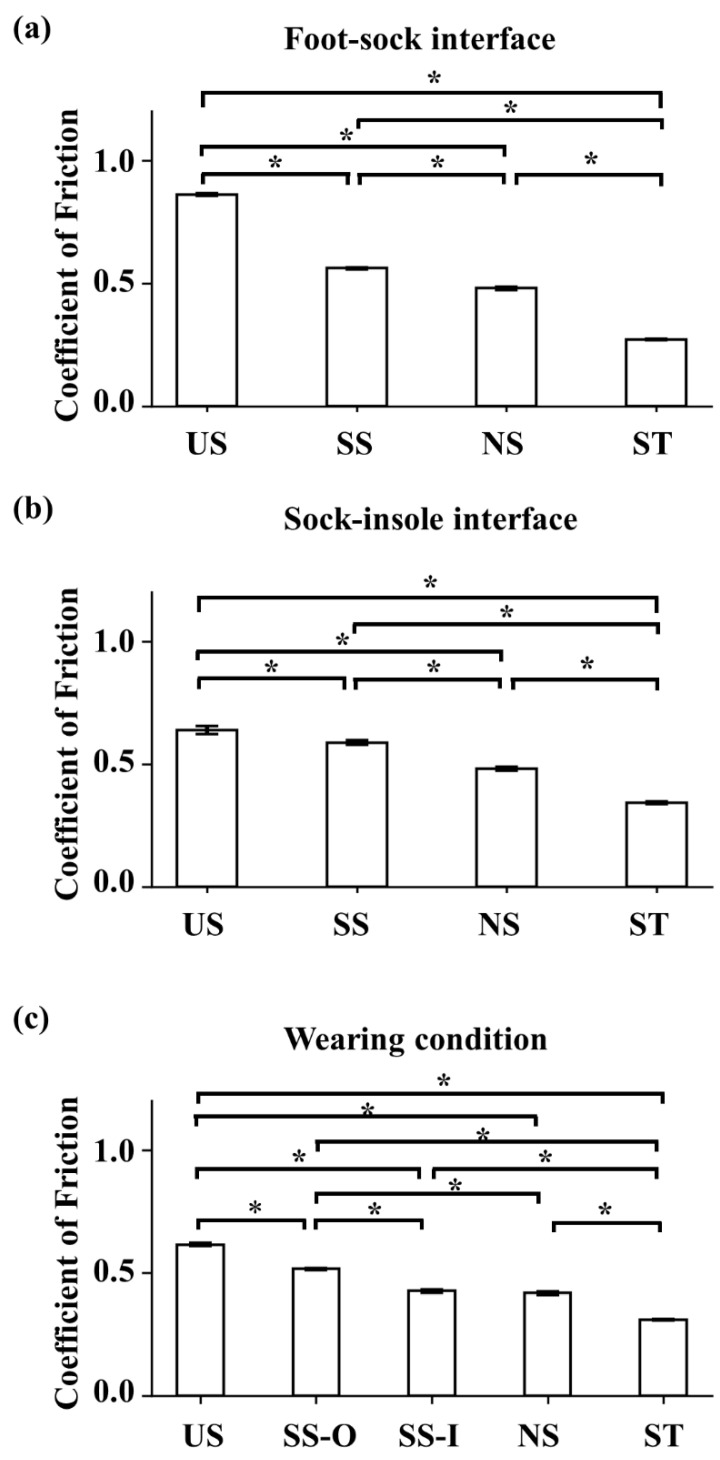
Static coefficient of friction measured by the ramp protocol (* significant difference in the COF values between socks). (**a**) static COF values at the foot-sock interface for the four socks (mean ± 1STD); (**b**) static COF values at the sock-insole interface for the four socks (mean ± 1STD; (**c**) static COF values at the sock-insole interface in the wearing condition (mean ± 1STD).

A one-way ANOVA revealed that there was a statistically significant effect of sock type on static COF values at the contact between the last and the inner part of socks (F(3,16) = 11299.9, *p* < 0.001; Figure 4a). Following post-hoc t-tests revealed significant differences in all the comparisons. The US socks had the largest COF values (0.861 ± 0.006) compared to those values found in the other socks (0.561 ± 0.004 for SS sock; 0.480 ± 0.006 for NS sock; 0.271 ± 0.003 for ST sock; *p* < 0.001 for all comparisons), followed by SS COF values that were significantly greater than in the NS (*p* < 0.001) and ST (*p* < 0.001). NS COF values were greater than ST socks (*p* < 0.001).

There was a significant main effect of sock type on static COF values (F(3,16) = 796.0, *p* < 0.001) at the sock-insole interface (Figure 4b). Following pair-wise comparisons showed that all the comparisons were significant. US socks had the largest COF values (0.639 ± 0.016) compared to those found in the other socks (0.480 ± 0.008 for NS sock; 0.589 ± 0.009 for SS sock; 0.342 ± 0.006 for ST sock; *p* < 0.001 for all comparisons). SS COF values were significantly greater than in NS (*p* < 0.001) and ST (*p* < 0.001) socks while NS COF values were greater than ST COF values (*p* < 0.001). 

The main effect of sock type on COF values was significant (F(4,20) = 2119.5, *p* < 0.001) in the wearing condition (Figure 4c). Following pair-wise comparisons revealed significant differences in all paired comparisons except the SS-I vs. NS comparison (*p* = 0.182). As found in the earlier two conditions, US socks showed the largest COF values (0.614 ± 0.007) compared to the other socks (0.417 ± 0.006 for NS sock; 0.515 ± 0.004 for SS-O sock; 0.426 ± 0.007 for SS-I sock; 0.310 ± 0.003 for ST; *p* < 0.001 for all comparisons). The SS-O COF values were significantly greater than in the NS (*p* < 0.001), SS-I (*p* < 0.001), and ST (*p* < 0.001). COF values of the SS-I and NS were significantly greater than in the ST condition (*p* < 0.001 for the two comparisons).

## 4. Discussion

The static coefficient of friction (COF) for socks was evaluated by a novel protocol using the ramp and last that enabled the measurement of friction at both foot-sock and sock-insole interfaces in a sock-wearing condition with minimal effects of the human variability. The measured COF values were reliable (almost perfect), and the suggested protocol successfully identified differences in frictional properties between socks.

### 4.1. The Reliability of Static COF Values Measured by the Ramp Protocol

The reliability of static COF values measured with the suggested ramp test was almost perfect [26]. In this study, static COF values ranged from 0.310 to 0.861 across the three ramp test conditions, which fell in the ranges reported in previous studies [7,8,9]. Between-trial variability values (standard deviation) across conditions ranged from 0.003 to 0.016 (0.7% to 2.5%), which was comparable with the range of COF values (less than 3%) measured by a mechanical tester [27]. The low between-trial variability represented that the COF values by the ramp protocol were highly repeatable between measurements.

### 4.2. The Ramp Protocol Could Be Used to Measure the Static COF for Socks

The ramp protocol successfully identified the difference in frictional properties between socks. Wearing socks have shown to make changes in the frictional properties at the foot-insole interface [4,28], suggesting the importance of separate measurements of COF at the skin-sock and sock-insole interfaces. The first ramp condition was designed to evaluate the static COF at the foot skin against the inner part of the socks. Previous studies have used animal skin [28], finite element modeling [4], and skin model including Pelite [7,29] instead of human participants (e.g., rubbing on the skin) for minimizing the effects of between-participant variability on their friction measurements. Similarly, we utilized a human skin model made of polyurethane with similar surface and mechanical properties to human skin [25]. The skin model was attached to the bottom of the last to set up the ramp condition. As expected, socks with non-slip materials (US and SS) produced greater friction at the skin-sock interface than regular socks (NS). The static COF value of the socks with polyurethane non-slip pads (US) was significantly greater than the COF value of the socks with silicone protrusions (SS), suggesting a superior non-slip function of the non-slip pads. Similar differences in the static COF values were found in the second ramp condition designed to measure the friction at the outer part of socks against the insole. The non-slip socks showed greater friction than did normal socks. The US socks had greater friction at the skin against the inner part of the sock (0.861) than at the outer part of the sock against the insole (0.639). The polyurethane non-slip pad was designed and manufactured to provide greater friction at both contact interfaces. The pad placement and pattern that appeared on the outer part were designed differently from those on the inner part (WE FOOT TECHNOLOGY, Seoul, Korea), which may explain the different frictional properties of the sock’s inner-outer parts. Stocking showed the lowest frictional property in both ramp conditions, consistent with findings in an earlier study [5].

Silicon protrusions may not be practical to increase friction when attached to the inner part of socks. In the third ramp condition, static COF values were measured at the sock-insole interface while the last wore socks. The US socks showed the highest friction value in the first two ramp conditions. However, there was no significant difference in the static COF value between socks with silicon protrusions at the inner part (SS-I) and socks without non-slip materials (NS). In contrast, socks with silicon protrusions at the outer part (SS-O) resulted in greater static COF values than the NS socks did. Those results may suggest that the silicon protrusions can increase friction at the sock-insole interface in the sock-wearing condition only when attached to the outer part of socks. Sock designers and manufacturers may need to consider the effects of different non-slip materials and attachment locations (e.g., inner or outer) when developing new socks with specific frictional properties.

### 4.3. The Ramp Protocol Using the Last Could Be a Time Efficent and Flexible Option for Measuring the Static COF Values for Socks

The protocol suggested in this study may replace the need of human participants and better reflect the stretchiness observed in a sock-wearing condition. Although the involvement of human participants enables the simulation of skin-fabric contacts found in reality [7,11,13,30], it also often requires significant efforts and time for recruiting and training participants prior to fiction measurements, and friction values measured with humans may be susceptible to human variability, including force production [18] and skin properties [30]. Further, previous studies have utilized a sample of fabric elongated at a designated length to replicate the stretchiness occurring in a sock-wearing condition, but the tension may not accurately represent the stretchiness. We suggested the ramp test protocol as a substitute for previous friction measurement methods involving human participants and mechanical testers for better addressing the significant efforts, and the effects of human variability and stretchiness. The observed high repeatability and ability to distinguish the differences in frictional properties suggest that the ramp protocol could be a reliable and time-efficient method for measuring the static COF for socks in a real-world situation.

The ramp tester with the last could efficiently perform other testing scenarios. Sock and shoe developers need to quickly measure the frictional properties of their prototypes while developing new socks or insoles targeting a specific population, such as patients with diabetes and athletes. For example, basketball players may need to wear socks with higher friction to produce acute movements such as cutting [31] while long-distance runners may wear socks with less friction for preventing blisters [32]. Acrylic was used to manufacture the ramp surface on which other types of socks (e.g., different non-slip pads, fabric compositions) can be attached easily so that the developers may use this protocol to manufacture socks specific to a situation. Further, other skin models [28,29] can be attached to the bottom of the last to examine the effects of different skin models on the COF values.

### 4.4. Study Limitations

The last weight may not represent the normal force applied to the feet in daily life and sports. The last weight was around 200 N (20 kg), lower than normal forces (1000 N to 2000 N) observed in sports requiring acute directional changes such as side-cut and turning tasks [31] and even in walking on a flat surface with a preferred speed (e.g., less than 1000 N) [33]. Previous studies have reported that greater applied weights (greater normal force) can reduce the coefficient of friction at the contact interface [10,34], suggesting that the findings in the current study may not be generalizable for estimating COF values with greater normal forces. However, the resultant weight was the maximum where the last could be stabilized for the ramp conditions. Further, the resultant weight was not lower than weights previous studies have commonly used (e.g., 7 N to 20.2 N) to measure coefficient of friction using mechanical testers [7,8,10]. A future study with a last that can hold greater weights may be necessary to examine the effects of different normal forces on static COF values of socks measured by the suggested ramp protocol.

## 5. Conclusions

This study proposed a novel protocol to measure the static coefficient of friction (COF) for socks in a sock-wearing condition with less effects of human variability. We utilized the ramp test and last to reduce the role of human participants and replicate the sock stretchiness observed in a sock-wearing condition. The reliability of static COF values was almost perfect, and the protocol successfully identified different frictional properties of socks. Socks with polyurethane non-slip pads (US) showed the highest COF values in all the three experimental tests (0.861, 0.639, and 0.614 in the foot-sock, sock-insole, and wearing condition, respectively). In contrast, stockings showed the lowest COF values (0.271, 0.342, and 0.310). Those results suggest that the static COF of socks can be measured in a similar situation to that of wearing socks by the protocol with fewer efforts and minimal effects of human variability. Other skin models or socks can be attached and tested, enabling sock and shoe developers to quickly evaluate the frictional properties of sock prototypes without spending much effort dealing with human participants. Thus, the suggested protocol could be a reliable and time-efficient option for measuring the static COF measurements for socks in an actual sock-wearing condition under reduced effects of human variability.

## Data Availability

The data measured in this study is fully available.

## Supplementary Materials

The following supporting information can be downloaded at: https://www.mdpi.com/article/10.3390/s22155525/s1. Table S1. Static COF values measured in the three experimental conditions.
